# Comprehensive clinical phenotyping of nitroglycerin infusion induced cluster headache attacks

**DOI:** 10.1177/0333102421989617

**Published:** 2021-02-20

**Authors:** Diana Y Wei, Peter J Goadsby

**Affiliations:** 1 Headache Group, Institute of Psychiatry, Psychology and Neuroscience, King’s College London, London, UK; 2 NIHR Wellcome Trust King’s Clinical Research Facility, King’s College Hospital, London, UK; 3 Department of Neurology, University of California, Los Angeles, CA, USA

**Keywords:** Cluster headache, nitroglycerin, cranial autonomic symptoms, non-headache symptoms, nitroglycerin headache, episodic cluster headache, chronic cluster headache

## Abstract

**Background:**

Nitroglycerin administration allows the study of cluster headache attacks in their entirety in a standardised way.

**Methods:**

A single-blind, placebo-controlled, cross-over study using weight-calculated intravenous nitroglycerin administration at 0.5 µg/kg/min over 20 minutes to study cluster headache attacks, including accompanying non-headache symptoms and cranial autonomic symptoms.

**Results:**

Thirty-three subjects with cluster headache were included in the study; 24 completed all three study visits. Nitroglycerin-induced attacks developed in 26 out of 33 subjects (79%) receiving unblinded nitroglycerin infusion, and in 19 out of 25 subjects (76%) receiving single-blinded nitroglycerin infusion, compared with one out of 24 subjects (4%) receiving single-blinded placebo infusion. Episodic cluster headache subjects had a shorter latency period to a nitroglycerin-induced attack compared to the chronic cluster headache (CCH) subjects (*U* = 15, z = −2.399, *p* = 0.016). Sixteen of nineteen episodic cluster headache (mean, 84%; 95% confidence interval, 66–100%) and 11 of 14 chronic cluster headache subjects developed a nitroglycerin-induced attack (79%, 54–100%) following the unblinded nitroglycerin infusion. Following the single-blinded nitroglycerin infusion, eight out of 13 episodic cluster headache (62%, 31–92%) and 11 out of 12 chronic cluster headache (92%, 73–100%) subjects developed nitroglycerin-induced attacks. Nitroglycerin induced non-headache symptoms in the majority of subjects receiving it: 91% in the open unblinded nitroglycerin visit and 84% in the single-blinded nitroglycerin visits, compared with 33% in the single-blinded placebo visit. Cranial autonomic symptoms were induced by nitroglycerin infusion, 94% in the open unblinded nitroglycerin visit and 84% in the single-blinded nitroglycerin visit, compared with 17% in the single-blinded placebo visit.

**Conclusion:**

Intravenous weight-adjusted nitroglycerin administration in both episodic cluster headache in bout and chronic cluster headache is effective and reliable in inducing cluster headache attacks, cranial autonomic symptoms and non-headache symptoms.

## Introduction

Due to the episodic nature of acute attacks of cluster headache, a standardised and reliable method for triggering attacks is necessary for their complete study. There have been several substances used to trigger attacks, including histamine (1,2), meta-chloro-phenylpiperazine (mCPP) (3) and more recently calcitonin gene-related peptide (CGRP) (4). Nitroglycerin (NTG) is the most frequently employed substance in human experimental modelling of cluster headache attacks, being used since 1953 (2) (Table 1).

**Table 1.** Review of studies using nitroglycerin to induce cluster headache attacks.

Study	Number of CH patients	NTG dose and route	Control	Placebo	Rate of induced CH attack	Latency period before onset of headache (range in minutes)
Peters 1953 (2)	14	1.3 mg sublingual	No, but compared with migraine, combination of CH and migraine, vasodilating head pain and tension headache	No, but compared with histamine and nicotinic acid	78.6%	Not reported
Ekbom 1968 (5)	38 ECH: 28 in bout, 15 out of bout (of which five were tested in bout as well)	1 mg sublingual	No	No	In bout ECH 100% (n = 28) Out of bout 0% (n = 15)	12–72
Barre 1982 (6)	10 CH: two CCH, nine ECH in bout	1 mg sublingual	No	No	100%	34–52 Mean 45.9
Drummond et al. 1984 (7)	29 CH	0.9 mg sublingual	No	No	76%	Within 90 min
Drummond et al. 1985 (8)	22 CH: 10 CCH, 12 ECH in bout	0.9 mg sublingual	Yes, 10 in the control group without headache	No	ECH 75% (n = 9) CCH 60% (n = 6)	9–90 Mean 44
Bogucki 1990 (9)	21 ECH in bout	1 mg sublingual	No	No	67% (n = 14)	30–65
Dahl et al. 1990 (10)	15 ECH in bout	1 mg sublingual	10 healthy controls	No	53% (n = 8) Controls: not reported	30–65
Hannerz et al. 1992 (11)	Eight ECH: five in bout and three out of bout	1 mg sublingual	No	No	In bout ECH 100% (n = 5)	Not reported
Fanciullacci et al. 1995 (12)	18 in bout ECH, 12 out of bout ECH	0.9 mg sublingual	No	No	In bout ECH 100% (n = 18) Out of bout ECH 0% (n = 12)	39–86
Fanciullacci et al. 1997 (13)	11 in bout ECH, eight out of bout ECH	0.9 mg sublingual	No	No	In bout ECH 100% (n = 11) Out of bout ECH 0% (n = 8)	18–50
Hsieh et al. 1996 (14)	Seven ECH: four in bout ECH, three out of bout	1 mg sublingual	No	No	In bout ECH 100% Out of bout ECH 0%	18–35
May et al. 1998 (15)	Nine CCH, eight out of bout ECH	1.0–1.2 mg spray	No	No	CCH 100% Out of bout ECH 0%	Not reported
Costa et al. 2000 (16)	15 CH: Six ECH, nine CCH	0.9 mg sublingual	No	No	ECH 33% (n = 2) CCH 78% (n = 7)	19–41
Costa et al. 2003 (17)	18 ECH in bout	0.9 mg sublingual	Yes, 12 healthy sex and age-matched	No	ECH 67% (n = 12) Controls 0%	19–41
Sances et al. 2004 (18)	42 CH: 29 ECH (11 out of bout), 13 CCH	0.9 mg sublingual	Yes, 53 healthy controls	No	In bout ECH and CCH 81% (n = 25 out of 31) Controls 7.5% developed headache in 7-hour post-NTG	Mean onset latency of 65.4 ± 54.7

Cluster headache patients suffer from recurrent episodes of severe unilateral pain in the trigeminal region; therefore, historically, the main emphasis has been centred around the pain onset of a cluster headache, and indeed this is often used as the indicator of the start of the attack. However, from clinical practice, there has been growing awareness of nonpainful symptoms preceding the onset of pain (19,20) and in recent years, the clinical phenotype of cluster headache has been further characterised by retrospective accounts (21–23) and prospective diaries (24,25).

The most frequent route of administration of NTG has been sublingual; however, the bioavailability of sublingual NTG is extremely variable (26). Therefore, we used a weight-calculated dose for intravenous infusion in a standardised single-blind, placebo-controlled study. NTG is a pro-drug of nitric oxide, with predominant actions on the cyclic guanylate phosphate (cGMP) pathway (27), and from pre-clinical studies there are indications of neural actions more centrally. This study is the first single-blind, placebo-controlled cross-over study using a weight-adjusted dose of nitroglycerin administered intravenously in cluster headache patients, thereby allowing comprehensive investigation of the various stages of the acute attack of cluster headache.

## Methods

### Subject selection and recruitment

The study was advertised on the UK cluster headache patient website OUCH (UK) (Organisation for the Understanding of Cluster Headache UK) (https://ouchuk.org/research/research-volunteers-needed) and in the tertiary Headache centre in King’s College Hospital, London. Subjects interested in participating would make contact via a dedicated research email. Subjects would then be screened for eligibility for the study via emails and a telephone call; those who met the criteria and were interested in participating were invited to attend the study visit. The study was approved by the London, City & East Research Ethics Committee (Ref Number 16/LO/0693). Data were collected from August 2016 until January 2019.

The subjects enrolled fulfilled the ICHD-3 beta criteria for cluster headache (28), and were between the ages of 18 and 60, with no significant previous medical history and no previous syncope or history of autonomic dysfunction. They had had a reliable response to high flow oxygen and/or subcutaneous sumatriptan during spontaneous attacks and normal brain neuroimaging. Women in child-bearing age were required to use reliable contraceptive methods during the study. Subjects were excluded if they were pregnant or breastfeeding, and who had any significant psychiatric disease, diagnosis of another primary headache type (other than migraine) or chronic pain syndrome, any medical history that would have contraindications to receiving NTG, use of preventive medication other than verapamil, or taking indomethacin for any reason, allergies to the medications used in the study or intolerance to high flow oxygen, and use of illicit drugs during the study.

### Study visits

The study comprised of three visits in total, with each visit separated by a minimum of 1 week. During the first visit, subjects consented and a full headache history with a physical examination of the neurological and cardiovascular systems was performed, along with recordings of the baseline blood pressure, lying and standing blood pressure, heart rate, oxygen saturation, weight and electrocardiogram. Female subjects were required to have a urinary pregnancy test before the start of the infusion.

### Open NTG infusion

If deemed eligible, subjects received an intravenous infusion of nitroglycerin at 0.5 µg/kg/min over 20 minutes. Subjects remained recumbent for 30 minutes before the infusion, and after the NTG infusion, subjects received 250 mL of 0.9% sodium chloride intravenously. The blood pressure and pulse were checked at the start of the infusion, and every 5 min during the infusion.

### Phenotypic characterisation

**Figure 1.**
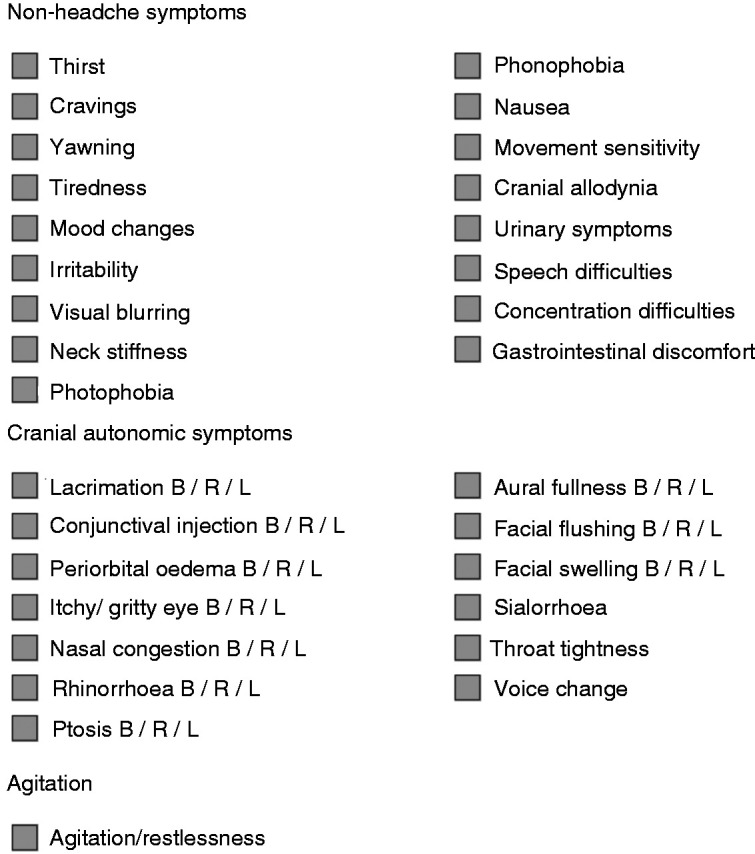
Questionnaire for non-headache and cranial autonomic symptoms. B: bilateral; R: right-sided; L: left-sided.

Every 5 minutes, subjects were asked to rate their pain level from a scale from no pain, mild, moderate to severe, where severe equates to their most severe attack, and they were asked a questionnaire of non-headache symptoms and cranial autonomic symptoms (CAS) (Figure 1). If the subject experienced pain, further details were obtained regarding the location and characteristic. Beyond the cluster headache attack, subjects were asked to report any other headache types and in particular NTG headache, which has been well described from migraine studies (29). The NTG headache was assessed for severity, phenotype, accompanying non-headache symptoms and duration. Subjects were actively encouraged to report any symptoms as and when they developed. Following the infusion, at 10 minute intervals, any developments in pain, non-headache symptoms and CAS were recorded for a total of 120 minutes after the infusion, based on the latency period reported from previous studies (Table 1).

### Single-blinded NTG and placebo infusions

**Figure 2.**
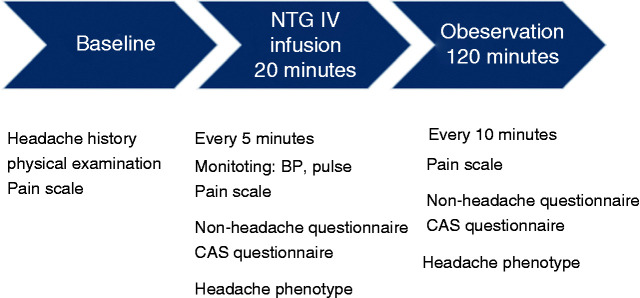
Outline of events from open unblinded nitroglycerin visit. BP: blood pressure; CAS: cranial autonomic symptoms; NTG: nitroglycerin; IV: intravenous.

During the two single-blinded visits, subjects either received intravenous nitroglycerin at 0.5 µg/kg/min over 20 minutes or an equal volume of 0.9% sodium chloride at the same rate over 20 minutes, followed by the same 120 minutes of observations (Figure 2). The sequence was pre-determined using the randomise function in Excel; however, the investigator was able to amend this in certain situations if the subject was episodic and due to finish their bout.

### Treatment of acute attacks

Acute treatment was administered at 20 minutes from the start of attack either with sumatriptan 6 mg subcutaneous injection (Imigran 6 mg/0.5 ml solution for injection pre-filled syringes, GlaxoSmithKline UK Ltd, or sumatriptan 6 mg/0.5 ml solution for injection pre-filled pens, Sun Pharmaceuticals Industries Europe B.V.) or with 15 L/min oxygen via a non-rebreather mask (O2Star™ non-rebreather oxygen mask M/L, Dräger). The visit was concluded only when the subject was pain free.

### Statistical analysis

The data was tabulated (Excel for Mac v 16.30), and descriptive statistics were performed to summarise data (SPSS Statistics version 26 for Mac and Excel). Mann-Whitney tests were performed on time until attack onset and time until CAS onset for those that developed NTG-triggered cluster headache attacks and CAS, comparing the differences in ECH with CCH subgroups. Kaplan-Meier survival graphs were used to examine the time until onset of event; that is, survival of no cluster headache attack status, or absence of CAS after NTG infusion for 140 minutes (20 minutes of infusion and 120 minutes of post-infusion observation), with log-rank statistical testing; *P* < 0.05 was considered significant. Graphs were made using SPSS and violin plots were made using GraphPad Prism 8 for Mac OS.

## Results

### Demography

**Figure 3.**
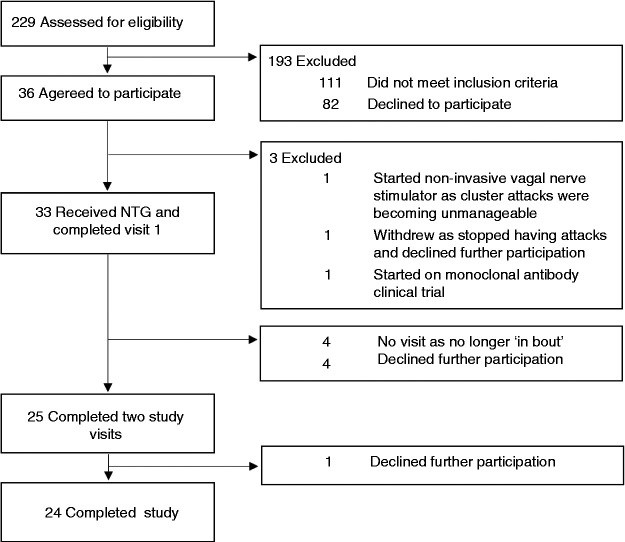
Subject numbers throughout the study.

A total of 229 subjects were contacted to check for eligibility, of which 33 were included in the study; 24 completed all three study visits (Figure 3). All patients fulfilled the ICHD-3 beta criteria for cluster headache, 19 episodic cluster headache in bout (58%) and 14 chronic cluster headache (42%) (Table 2). The majority were male (n = 24, 73%) with a male: female ratio of 3:1. The average age was 41 (SD 10), with the mean age of females (34 years, SD 9) younger than in males (44 years, SD 9) in this cohort. Subjects had had the condition for a median of 9 years (IQR 5–16).

**Table 2.** Subject characteristics.

Subject	Age	Sex	Subtype	Side of attacks	Time since first attack (years)	Average attack frequency (per day)	Average attack duration when untreated (mins)	Average bout duration (weeks)	Verapamil (total daily dose in mg)
1	55	M	Chronic	Right	5	2	45	–	480
2	28	F	Chronic	Right	12	7	150	–	–
3	40	M	Episodic	Right	20	3	150	8	–
4	38	F	Episodic	Left	12	2	120	4	–
5	53	M	Chronic	Left	15	4	35	–	960
6	48	M	Episodic	Left	25	5	52.5	5	480
7	23	M	Episodic	Left	8	2	120	10	–
8	43	M	Chronic	Right	9	1	40	–	–
9	43	M	Episodic	Right	13	3	60	5	240
10	44	M	Episodic	Right	27	6	45	6	–
11	47	M	Episodic	Right	3	1	105	20	–
12	48	M	Episodic	Left	26	3	180	10	560
13	40	M	Episodic	Right	5	3	60	8	–
14	50	M	Episodic	Right	10	2	105	8	–
15	56	M	Episodic*	Right	24	1	45	–	–
16	35	M	Episodic	Left	7	2	150	6	240
17	58	M	Episodic	Left	3	1	135	24	–
18	43	M	Chronic	Left	4	2	60	–	–
19	32	M	Episodic	Left	3	3	50	12	600
20	49	F	Episodic	Right	8	5	50	9	–
21	49	M	Chronic	Right	35	2	90	–	–
22	57	M	Chronic	Right	20	5	65		–
23	40	F	Chronic	Left	4	1.5	120	–	640
24	44	M	Chronic	Right	6	2	90	–	720
25	23	F	Episodic	Right	9	2.5	90	6	–
26	40	M	Chronic	Left	17	1.5	180	–	–
27	20	F	Chronic	Right	6	1.5	150	–	–
28	31	F	Episodic	Right	14	2	75	11	–
29	35	M	Episodic	Left	9	2	90	3	–
30	38	F	Chronic	Left	6	1.5	180	–	240
31	49	M	Episodic	Left	0.5	1.5	37.5	21	400
32	35	F	Chronic	Left	4	0.3	30	–	600
33	32	M	Chronic	Left	10	6	60	–	–

M: male; F: female.

*Subject was chronic but became episodic during study with no attacks in 6 months.

### Clinical phenotyping of subjects by history

#### Pain features

There was a near-equal split between the laterality of attacks; 17 subjects had right-sided attacks (52%), and 16 subjects had left-sided attacks (49%). The median number of attacks per day was two (IQR 1.5–3) lasting a median of 90 minutes (IQR 50–128). Verapamil was the only preventive allowed on the study; 12 subjects (36%) were on verapamil ranging from 240 mg to 960 mg per day.

#### Migraine, accompanying features and non-headache symptoms in spontaneous attacks

In this cohort, 58% (n = 19) had a concurrent migraine (28): five had migraine with aura, of which four had visual aura and one had facial sensory aura. Of the subjects who did not meet the diagnostic criteria for migraine (28) (n = 14, 42%), the majority had either migraine markers and/or a family history of migraine. Migraine markers were defined if subjects experienced: childhood cyclic nausea and vomiting, motion sickness, cold stimulus-induced headache, hangover headache and jetlag. Four subjects did not have migraine markers, and three subjects (9%) did not have either migraine marker or a family history of migraine. Subjects (n = 24, 73%) reported accompanying symptoms with their spontaneous attacks, the most common symptoms being cranial allodynia (n = 15), photophobia (n = 15), nausea (n = 11) and phonophobia (n = 10).

#### Non-headache attack symptoms

The majority reported non-headache symptoms in the lead up to their spontaneous cluster headache attacks (n = 27, 82%), with the commonest symptoms being concentration difficulties (n = 18), mood changes (n = 15), neck stiffness (n = 13) and yawning (n = 8). The median onset time of symptoms was 10 minutes (IQR 2–30) before the onset of pain. Although most symptoms start before the onset of pain, five subjects only reported developing the symptoms during the attack.

Twenty-eight subjects reported non-headache symptoms following their cluster headache attacks (85%), the commonest symptoms being tiredness (n = 27), mood changes (n = 13), concentration difficulties (n = 12), and neck stiffness (n = 7). The median duration of symptoms was 120 minutes (IQR 53–315).

#### Attack triggers

The most commonly reported triggers for spontaneous attacks were alcohol ingestion (n = 22), followed by changes in temperature (n = 12), strong smells such as from nail varnish, paint (n = 11) followed by disrupted sleep (n = 4) and exercise (n = 3). Other triggers mentioned were stress (n = 2), caffeine (n = 2), changing time zones (n = 1), let down from stress (n = 1), being in a pressurised cabin on an airplane (n = 1), vardenafil (n = 1), and dehydration (n = 1). All subjects reported attacks within a few hours of alcohol ingestion, no one reported a next-day effect.

### Clinical phenotyping of open NTG visit

NTG triggered cluster headache-like attacks (n = 26, 79%), CAS (n = 31, 94%) and agitation (n = 24, 73%) during the open unblinded NTG visit. In the episodic cluster headache group, 16 out of 19 developed an NTG-induced attack (mean, 84%; 95% CI, 66–100%) and in the chronic cluster headache group, 11 out of 14 developed an NTG-induced attack (79%, 54–100%). Five out of the 31 subjects developed CAS, but did not develop an attack; of these subjects, three developed mild generalised pain, one developed restless legs, and one developed CAS on the contralateral side to the subject’s spontaneous attack phenotype, with a generalised headache. These cases were excluded.

The median onset time for CAS to develop from the start of the NTG infusion was 31 minutes (IQR 13–46). The median onset for an NTG-induced attack was 30 minutes (IQR 20–39) and the median time to maximum pain, if reached, was 51 minutes (IQR 39–70). The most common first cranial autonomic symptoms brought on by NTG infusion were nasal congestion (n = 10), conjunctival injection (n = 5), lacrimation (n = 4) and periorbital oedema (n = 3).

### Clinical phenotyping of single-blinded NTG and placebo visit

**Figure 4.**
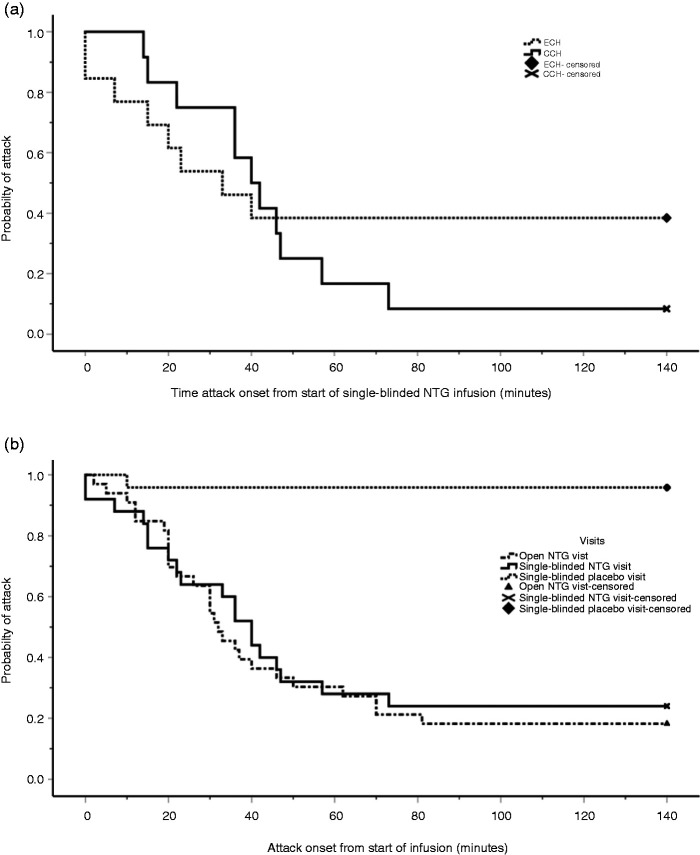
(a) Kaplan-Meier graph comparing time until cluster headache attack between the eight episodic cluster headache (ECH) and the 11 chronic cluster headache (CCH) subjects during single-blinded nitroglycerin (NTG) infusion, from the start of the infusion in minutes, log-rank *P* = 0.534. (b) Kaplan-Meier graph comparing time until cluster headache attack onset between the open unblinded (n = 33) and single-blinded (n =25) nitroglycerin (NTG) infusions with single-blinded placebo infusions (n = 24). Log-rank *P* = 0.000.

During the single-blinded visits, NTG induced attacks in 19 subjects (76%); three subjects did not have an attack (12%), two subjects developed migraine-like headache (8%) and one subject developed unilateral mild pain without CAS or agitation (4%) (Table 3). In the placebo group, only one developed an attack (4%), two subjects developed unilateral mild pain without CAS or agitation (8%) and two subjects experienced short-lived unilateral CAS (8%). Of the subjects receiving NTG, eight out of 13 ECH (mean, 62%; 95% CI, 31%-92%) and 11 out of 12 CCH (92%, 73–100%) developed NTG-induced attacks (Figure 4(a)).

**Table 3.** Subject characteristics and comparison between spontaneous attack and triggered attack from single-blinded nitroglycerin visit, M = male, F = female, ECH = episodic cluster headache, CCH = chronic cluster headache, L = Left, R = Right, Y = Yes, N = No, VRS = verbal rating scale, mod = moderate, O2 = high flow oxygen via non-rebreather mask, suma = sumatriptan.

Subject	Age	Sex	Subtype	Migraine	Verapamil (total daily dose in mg)	Spontaneous attack				Triggered attack- single-blinded NTG visit						
						Laterality	Severity	CAS	Agitation	Laterality	Severity/VRS	CAS	Agitation	Feels like spontaneous attack	Treatment	Last spontaneous attack (days)
1	55	M	CCH	N	480	R	9/10	Conjunctival injection, nasal congestion, lacrimation. 3 months of R Horner’s syndrome in 2007, now resolved	Y	R	10/10 VRS 3	Conjunctival injection, nasal congestion, lacrimation, periorbital oedema	Y	Y	O2	158
2	28	F	CCH	Y	–	R	10/10	Periorbital oedema/ptosis, nasal congestion, dry eye then lacrimation	Y	R	10/10 VRS 3	Periorbital oedema, nasal congestion, facial flushing	Y	Y	O2	128
3	40	M	ECH	N	–	R	10/10	Lacrimation, rhinorrhoea, ptosis	Y	R	10/10 VRS 3	Lacrimation, conjunctival injection, periorbital oedema, nasal congestion, aural fullness and facial droop	Y	Y	Suma	1
4	38	F	ECH	Y	–	L	10/10	Lacrimation, conjunctival injection, periorbital oedema, nasal congestion, ptosis, facial flushing	Y	No attack, developed generalised pain 4/10			Y	N	N/A	1
5	53	M	CCH	N	960	L	10/10	Rhinorrhoea, ptosis	Y	L	10/10 VRS 3	Conjunctival injection, eye grittiness, lacrimation	Y	Y	O2	4
6	48	M	ECH	N	480	L	8/10	Nasal congestion, lacrimation	Y	Did not attend						
7	23	M	ECH	Y	–	L	10/10	Lacrimation, conjunctival injection, nasal congestion, ptosis, facial flushing	Y	Did not attend						
8	43	M	CCH	N	–	R	10/10	Nasal congestion, rhinorrhoea, lacrimation, facial flushing	Y	R	10/10 VRS 3	Nasal congestion, conjunctival injection, eye grittiness	Y	Y	Suma	<1
9	43	M	ECH	N	240	R	10/10	Lacrimation, conjunctival injection, facial flushing, ptosis, facial droop	Y	Did not attend						
10	44	M	ECH	N	–	R	10/10	Nasal congestion, ptosis, periorbital oedema, lacrimation, flushing	Y	No attack, L mild hangover like headache, with nasal congestion. After study visit felt nauseous and vomited, then slept, headache free at 7pm			N	N	O2- no response	<1
11	47	M	ECH	N	–	R	Severe attacks 10/10	Nasal congestion, aural fullness	Y	R	7/10 VRS 2/3 moderate/ severe	Nasal congestion, conjunctival injection, aural fullness	Y	Y	Suma	13
12	48	M	ECH	N	560	L	10/10	Lacrimation, nasal congestion, periorbital oedema	Y	Did not attend						
13	40	M	ECH	Y	–	R	10/10	Nasal congestion, ptosis, conjunctival injection, lacrimation, throat tightness, rhinorrhoea	Y	No attack, bilateral mild headache 4/10 with nasal congestion			Y, brief (7 mins)	N	N/A	19
14	50	M	ECH	Y	–	R	10/10	Lacrimation, periorbital oedema, nasal congestion, rhinorrhoea	Y	Did not attend						
15	56	M	ECH	Y	–	R	10/10	Lacrimation, rhinorrhoea, facial flushing, gritty eyes, conjunctival injection	Y with severe attacks	Did not attend						
16	35	M	ECH	Y	240	L	10/10	Nasal congestion, conjunctival injection, ptosis, lacrimation, rhinorrhoea	Y	Not attack, milder than a shadow and no CAS			N	N	N/A	16
17	58	M	ECH	Y	–	L	10/10	Nasal congestion, rhinorrhoea, lacrimation, aural fullness, throat swelling	Y	L	7/10 VRS 2–3 (mod-severe)	Nasal congestion, rhinorrhoea, voice change, lacrimation	Y	Y	Suma	1
18	43	M	CCH	Y	–	L (70%)	Mild 6/10, severe 10/10	Nasal congestion, periorbital oedema, aural fullness, facial flushing	Y	L	Mild, VRS 1	Nasal congestion	Y	Y- shadow	O2	7
19	32	M	ECH	Y	600	L	10/10	Lacrimation, conjunctival injection, facial flushing	Y	L	7–8/10 VRS 2–3 (mod-severe)	Lacrimation, eye grittiness, nasal congestion, facial flushing	Y	Y	O2	<1
20	49	F	ECH	Y	–	R	10/10	Ptosis, periorbital oedema, lacrimation, nasal congestion, rhinorrhoea, (aural fullness with severe attacks)	Y	R	6–7/10 VRS 2 mod	Nasal congestion, periorbital oedema, lacrimation, conjunctival injection	Y	Y	O2	7
21	49	M	CCH	Y	–	R	4–10/10	Facial flushing, nasal congestion, ptosis, lacrimation, rhinorrhoea, conjunctival injection	Y	L	8/10 VRS 2–3 (mod-severe)	Facial flushing, lacrimation, periorbital oedema, conjunctival injection	Y	Y, but on L rather than R	Suma	<1
22	57	M	CCH	N	–	R	10/10	Lacrimation, conjunctival injection, nasal congestion, rhinorrhoea	Y	No attack, no pain and no CAS			N	N	N/A	26
23	40	F	CCH	Y	640	L	10/10	Lacrimation, conjunctival injection, periorbital oedema, rhinorrhoea, ptosis, aural fullness	Y	Did not attend						
24	44	M	CCH	N	720	R	10/10	Lacrimation, conjunctival injection, periorbital oedema, facial flushing	Y	Did not attend						
25	23	F	ECH	Y	–	R	6–10/10	Nasal congestion, lacrimation, periorbital oedema. Rhinorrhoea and facial flushing with severe attacks	Y	R	7/10 VRS 2 (moderate)	Nasal congestion, periorbital oedema	Y	Y	O2	3
26	40	M	CCH	Y	–	L	Shadow 4/10, daytime attacks 6–7/10, full attack 10/10	Ptosis, aural fullness, lacrimation, conjunctival injection, periorbital oedema, nasal congestion	Y	L	8.5–9/10 VRS 3 severe	Nasal congestion, periorbital oedema, lacrimation	Y	Y	Suma	3
27	20	F	CCH	N	–	R	8–9/10	Nasal congestion, lacrimation, rhinorrhoea, ptosis, aural fullness	Y	R	8/10 VRS 3 severe	Nasal congestion, periorbital oedema	Y	Y	O2	<1
28	31	F	ECH	Y	–	R	10/10	Nasal congestion, periorbital oedema, rhinorrhoea, ptosis, aural fullness	Y	R	2/10 VRS 1 (mild)	Nasal congestion, facial swelling	Y	Y, but milder	Suma	3
29	35	M	ECH	N	–	L	Max pain 10/10	Nasal congestion, periorbital oedema, lacrimation, voice change (croaky). Facial flushing with severe attacks and rhinorrhoea after attack	Y	L	6–7/10 VRS 2 (moderate)	Nasal congestion, lacrimation, conjunctival injection, voice change	Y	Y	Suma	<1
30	38	F	CCH	Y	240	L	4–10/10	Lacrimation, rhinorrhoea, conjunctival injection, facial flushing. Ptosis with severe attacks	Y	L	6–7/10 VRS 2 (moderate)	Aural fullness	Y	Y	Suma	1
31	49	M	ECH	Y	400	L	10/10	Nasal congestion, lacrimation, conjunctival injection, rhinorrhoea, ptosis, aural fullness. Prominent miosis after attack	Y	No attack, whole head felt heavy			N	N	N/A	10
32	35	F	CCH	N	600	L	4/10 (on verapamil), 7/10 average	Periorbital oedema, eye grittiness, facial flushing, lacrimation	Y	L	7/10 VRS 2–3 (mod-severe)	Nasal congestion, conjunctival injection, facial flushing, lacrimation	Y	Y	Suma	1
33	32	M	CCH	Y	–	L	Severe attacks 10/10	Lacrimation, ptosis, nasal congestion, conjunctival injection, ptosis, facial flushing, eye grittiness	Y	L	6/10 VRS 2 (mod-severe)	Lacrimation, nasal congestion, ptosis	Y	Y	Suma	<1

The median onset time to an induced attack was 33 minutes (IQR 15–42) from the start of the single-blinded NTG infusion, and in the single-blinded placebo visit only one subject developed an attack; the onset time was 10 minutes. On comparison of the attack onset time for the unblinded NTG, single-blinded NTG and single-blinded placebo visits, there was a difference between the placebo and NTG visits (*P* = 0.000, Figure 4(b)).

### NTG headache

The majority of subjects developed an NTG headache (n = 28, 85%) following the NTG infusion in the unblinded open visit, of which 27 subjects had a migraine diagnosis (n = 18), had migraine markers (n = 10) and a family history of migraine (n = 18); only one subject did not have migraine, migraine markers or a family history of migraine. The median time from the start of NTG infusion was 3 minutes (IQR 2–6), and the median duration of NTG headache was 26 minutes (IQR 14–41).

Of the 25 subjects that went on to the single-blinded NTG visits, 18 (72%) developed NTG headache following NTG infusion, of which all had either migraine (n = 11), migraine markers (n = 7) or family history (n = 10). The median time of NTG headache onset from the start of NTG infusion in the blinded visit was 4 minutes (IQR 3–8), with a median duration of 26 minutes (IQR 9–45).

In the placebo visit, three of the 24 subjects (13%) developed NTG headache, median onset time was 13 minutes (range 1–18), and the median duration was 10 minutes (range 3–16).

### Non-headache symptoms

The majority of subjects reported non-headache symptoms in both the open unblinded NTG visit (n = 30, 91%) and the single-blinded NTG visit (n = 21, 84%) visits. In the single-blinded placebo visit, eight subjects (33%) reported non-headache symptoms. The most common non-headache symptoms from the open unblinded NTG visit were neck stiffness, photophobia, thirst and allodynia. The most common non-headache symptoms from the single-blinded NTG visit were neck stiffness, yawning, thirst and photophobia (Table 4). The median number of non-headache symptoms reported in the open unblinded NTG visit was three (IQR 2–5), and from the single-blinded NTG visit was three (IQR 2–6), compared with two (IQR 2–3) on-headache symptoms in the single-blinded placebo group.

**Table 4.** Frequencies of non-headache symptoms reported during each visit.

Non-headache symptom	Open unblinded NTG visit (n = 33)	Single-blinded NTG visit (n = 25)	Single-blinded placebo visit (n = 24)
Thirst	15	10	3
Craving	0	0	0
Yawning	8	12	4
Tiredness	4	3	0
Mood changes	5	4	1
Visual blurring	4	3	1
Neck stiffness	21	15	3
Irritability	3	3	0
Photophobia	18	10	1
Concentration difficulties	6	4	1
Phonophobia	4	6	0
Urinary symptoms	0	0	0
Speech disturbances	0	0	0
Nausea	6	3	0
Gastrointestinal discomfort	0	0	0
Movement sensitivity	5	5	1
Allodynia	9	8	2

In the open unblinded NTG visit, the median onset time of non-headache symptoms from the start of the infusion was 9 minutes (IQR 4–17) with the median maximum duration of the symptoms being 50 minutes (IQR 24–64), the duration is calculated from the first symptom until the end of the last symptom. In the single-blinded NTG visit, the median onset time was 5 minutes (IQR 3–9) from the start of the infusion, and the median maximum duration of symptoms was 59 minutes (IQR 35–76). The outlier was a subject who developed allodynia and neck stiffness during their attack. For those that developed non-headache symptoms during the single-blinded placebo visit, the median onset time was 9 minutes (IQR 2–9), with a median duration of 16 minutes (IQR 6–34).

### Cranial autonomic symptoms

NTG triggered CAS in the majority of the subjects during the open unblinded NTG visit (n = 31, 94%). In all, five subjects out of the 31 (19%) developed unilateral CAS; however, they did not have a cluster headache attack. Two subjects had CAS with generalised headache, one had CAS without any pain, one subject developed CAS on the contralateral side to their attacks with mild generalised pain, and one subject with CAS developed restless leg symptoms, but no cluster headache attack. Of the subjects who developed unilateral CAS and cluster headache attack (n = 26, 79%), the median number of CAS was four (IQR 2–5), with the most common CAS being nasal congestion (n = 21), lacrimation (n = 16) and periorbital oedema (n = 15) (Table 5).

**Table 5.** Frequencies of cranial autonomic symptoms reported in subjects who had attacks following NTG infusion.

Cranial autonomicsymptoms	Open unblinded NTG visit, total number of attacks (n = 26)	Single-blinded NTG visit, total number of attacks (n = 19)
Lacrimation	16	11
Conjunctival injection	12	9
Periorbital oedema	15	8
Eye grittiness/itchiness	3	3
Nasal congestion	21	16
Rhinorrhoea	2	1
Ptosis	4	1
Aural fullness	5	2
Facial flushing	7	4
Sialorrhoea	1	0
Throat swelling	2	0
Voice change	4	2
Facial swelling	3	1

**Figure 5.**
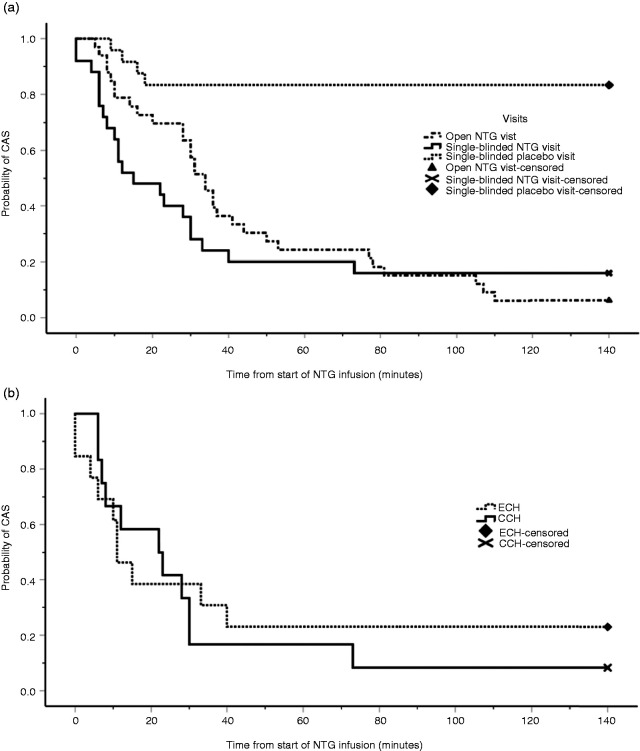
(a) Kaplan-Meier graph showing time until onset of cranial autonomic symptoms (CAS) following open unblinded (n = 33) and single-blinded NTG (n = 25) infusions compared with the single-blinded placebo infusion (n = 24). Log-rank *P* = 0.000. (b) Kaplan-Meier graph showing the time until CAS onset in the single-blinded NTG visit, comparing episodic cluster headache (ECH) with chronic cluster headache (CCH). Log-rank *P* = 0.740.

Similarly, NTG brought on CAS in the majority of subjects during the single-blinded NTG visit (n = 21, 84%), of which two were not accompanied by a cluster headache attack, compared with four out of 24 in the single-blinded placebo group developing CAS (Figure 5(a)). One of the subjects from the single-blinded NTG group had a generalised headache similar to a hangover headache with unilateral CAS, and the other subject had a migraine-like headache with CAS. Of the 19 subjects (76%) that had unilateral CAS and a cluster headache attack, the median number of symptoms was three (IQR 2–4) and the most frequent symptoms were nasal congestion (n = 16), lacrimation ( n = 11) and conjunctival injection (n = 9) (Table 5). In comparing the time until all CAS onset between ECH and CCH after blinded NTG infusion, there was no difference between the two groups (log-rank *P* = 0.740; Figure 5(b)).

The median time until CAS onset for those that developed cluster headache attacks was 31 min (IQR 13–46) in the open unblinded NTG visit and 12 min (IQR 6–30) in the single-blinded NTG visit. Subjects sequentially reported CAS development.

During the single-blinded placebo visit, four subjects (17%) developed unilateral CAS, of which one subject developed a spontaneous attack, and three developed CAS but no pain. The median number of symptoms reported in the placebo group was two (IQR 1–2) and the symptoms reported were nasal congestion (n = 2), conjunctival injection (n = 1), periorbital swelling (n = 1), rhinorrhoea (n = 1) and aural fullness (n = 1).

#### Agitation

Nitroglycerin brought on agitation in the majority of subjects, open unblinded NTG visit (n = 24, 73%) and single-blinded NTG visit (*n*= 20, 80%), compared with none in the single-blinded placebo visit.

#### Non-headache symptoms post-attack

The non-headache symptoms were not studied here, as all attacks were treated with sumatriptan or oxygen after 20 minutes, thus altering the natural progression and development of symptoms.

### Episodic cluster headache compared with chronic cluster headache

For the subjects that developed an NTG-induced attack following the single-blinded NTG infusion, the time until NTG-induced attack was shorter in the episodic cluster headache group compared with the chronic cluster headache group (*U* = 15, z = −2.399, *P* = 0.016). The time until CAS onset, in the subjects that developed CAS following single-blinded NTG infusion was not different between episodic cluster headache and chronic cluster headache groups (*U* = 38, z = −1.2000, *P* = 0.230).

### Effect of verapamil on NTG triggering

There were seven subjects on verapamil that attended the single-blinded NTG visits, of which two did not develop NTG-triggered cluster headache attacks (29%). Using χ^2^, there was no difference between verapamil and NTG triggering (χ^2^ (df = 1) = 0.111, *P* = 0.739).

### Effect of migraine on NTG-triggered attacks

Within the cohort that attended the single-blinded NTG visits, 15 had migraine (60%). There was no difference between the number of non-headache symptoms (*U* = 67, z = −0.449, *P* = 0.654) or number of CAS (*U* = 57, z = −1.019, *P* = 0.308), between the subjects with migraine and those without migraine. Furthermore, there was no association between subjects with migraine and NTG triggering (χ^2^ (df = 1) = 0.146, *P* = 0.702); similarly, there was no association between subjects with migraine and development of NTG headache (χ^2^ (df = 1) = 0.033, *P* = 0.856).

## Discussion

This study demonstrates that weight-calculated intravenous NTG effectively triggers cluster headache attacks in both chronic cluster headache and episodic cluster headache subjects within bout when compared with placebo. The approach is reliable in terms of triggering a fully-featured attack that allows careful observation of symptoms and their development during acute attacks of cluster headache.

The benefit of weight-calculated intravenous NTG compared to intranasal and sublingual administration is that this is more reliable and has more stable bioavailability. The median time until cluster headache attack onset was 30 minutes (open unblinded NTG visit) and 33 minutes (single-blinded NTG visit), with the overall maximum onset time of 81 minutes. Subjects who did not develop an attack during the 140 minutes of observation were asked to report if they developed an attack later on that day, and none did. The median onset time is similar to the reported latency period reported in the literature (Table 1). The minimum onset time for NTG-induced attacks was 2  minutes, and this is less than reported from the sublingual route, where the shortest time until onset time was 9 minutes (8); this is expected, given the intravenous route has a half-life of 2.3–2.8 minutes (30,31). Two subjects started experiencing mild attack pain before the start of the NTG infusion, and within the placebo group we observed one subject who developed a spontaneous attack. Spontaneous attacks could be anticipated given the subjects are within bout; there were three subjects with only short-lasting CAS symptoms and two subjects who experienced a mild pain without CAS, similar to a shadow.

**Figure 6.**
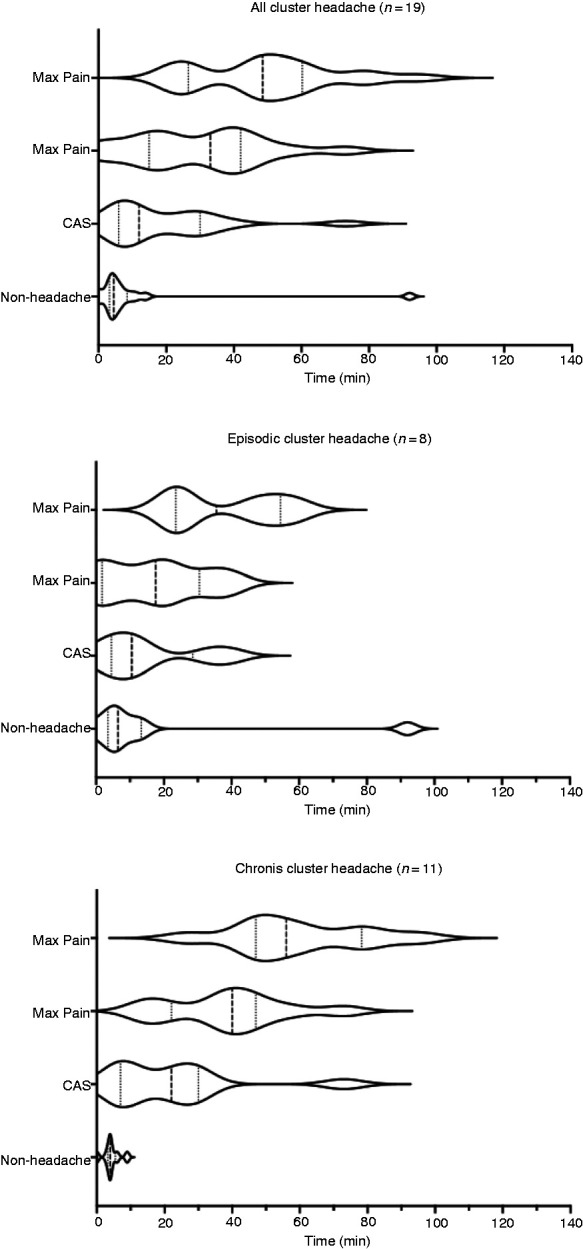
Violin plots of the stages of cluster headache attack from the 19 subjects that developed cluster headache attacks and subdivided by chronicity. The timeline starts with 20 minutes of single-blinded nitroglycerin (NTG) infusion, followed by 120 minutes of post-infusion observation. CAS = cranial autonomic symptoms.

This study delineates the development of the various stages of an NTG-induced cluster headache attack, including the non-headache symptoms and CAS (Figure 6). The majority of subjects developed NTG headache shortly after the infusion started. This headache was generalised, mild and progressive in nature, predominantly in the bi-occipital and bi-temporal regions. Subjects described it as pressure-like or band-like ache, and they reported it to be distinct from their cluster headache attacks; if there were an overlap, the cluster headache attack would clearly ramp up and supersede the NTG headache. It is difficult to disentangle whether NTG headache developed because there was a high proportion of subjects with either a migraine diagnosis, migraine marker or family history of migraine. However, from a previous study with 25 healthy volunteers without a migraine diagnosis, 16 (64%) developed NTG headache following 0.5 mg NTG sublingual administration (32).

Following the NTG headache, the majority of subjects reported non-headache symptoms in the lead-up to the onset of the pain from the attack and in some subjects, non-headache symptoms accompanied their attacks. The non-headache symptoms included homeostatic symptoms (thirst, cravings, yawning, frequency of urination), fatigue/cognitive symptoms (concentration difficulty, fatigue, memory impairment, mood changes, irritability) and sensitised sensory symptoms (neck stiffness, photophobia, phonophobia, osmophobia, nausea), as are reported in the premonitory phase of migraine (33). The most common symptoms were neck stiffness, photophobia and thirst. During their spontaneous attacks, subjects reported that non-headache symptoms would precede the attack at a median of 10 minutes (IQR 2–30) before the onset of pain, although it is known that there are discrepancies between retrospective and prospective reporting (25). Comparing the symptoms observed from NTG-induced attacks in this study with the prospective observational questionnaire study (24), the most reported general symptoms in the pre-pain phase in this study were concentration difficulties, photophobia and mood changes. Patients in the observational study also reported general symptoms during their attacks and postictal symptoms similar to the symptoms seen during the NTG-induced attacks and the reported symptoms by the subjects in this study during the postdrome phase of their spontaneous attacks. Non-headache symptoms such as photophobia, phonophobia, and localised allodynia were often reported during the attacks, as has been reported previously (21,34). In one semi-structured questionnaire study, they found a high proportion of cluster headache patients (73.2%) reported phonophobia or photophobia with their attacks (35), cluster headache patients with migraine did not more frequently report these symptoms compared with those without co-existing migraine. Furthermore, allodynia was more often reported in cluster headache patients with migraine. From pre-clinical studies, high dose NTG causes a sustained increase in spontaneous firing of Aδ and C-fibre trigeminal neurons (36), which migraine biology may unmask.

Similar to the prospective questionnaire study by Snoer and colleagues (24), in some subjects CAS presented before the onset of the attack pain (Figure 6). In this study, the median onset time for CAS was 31 minutes (IQR 13–46) in the open unblinded NTG visit and 12 minutes (IQR 6–30) in the single-blinded NTG visit, whereas the onset of NTG-induced attack was 30 minutes (IQR 20–39) in the open unblinded visit and 33 minutes (IQR 15–42) in the single-blinded NTG visit. From the open unblinded NTG visit, 38% presented with CAS before the attack, 15% presented with CAS with the onset of the attack, and from the single-blinded NTG visit, 58% presented with CAS before the attack and 16% presented with CAS with the onset of the attack.

Vollesen and colleagues used calcitonin gene-related peptide (CGRP) infusion to trigger cluster headache attacks, finding CGRP induced 50% of subjects with CCH and 89% for ECH subjects in bout (4). In our study, NTG induced attacks in 79% of the CCH subjects and 84% in ECH subjects in bout, in the open unblinded NTG visits, and 92% CCH and 62% ECH in bout in the single-blinded NTG visits. There are some differences in the number of chronic cluster headache subjects on verapamil between the studies; in this study, 33% of CCH subjects were on verapamil and in the study by Vollesen and colleagues, 57% of CCH subjects were on verapamil (of which one was on both verapamil and lithium) and one was on 4 mg melatonin. Vollesen and colleagues proposed that the CCH patients who did not develop an attack following CGRP infusion had a lower median attack frequency in the preceding 30 days prior to the study compared to the CCH patients that did develop an attack. However, in this study, subjects reported having their last attack as long ago as 128 and 158 days and still had NTG-induced attacks (Table 3).

NTG is a pro-drug of nitric oxide (NO) and has effects on blood vessel dilation through mechanisms of the NO-cyclic guanylate phosphate (cGMP) pathway (27). However, the vasodilatory effects of NTG are not sufficient to explain the cluster headache attack that occurs after a latency and the central features associated with the attack. Indeed, experimental studies demonstrate that NTG has central effects (37,38). Interestingly, in studies where NO donors were administered *in vivo* and *in vitro*, there was a local release of CGRP (39–42). The difference in the rate of cluster headache attacks induced by NTG and CGRP in ECH and CCH subjects could reflect in part the underlying biological processes and systems that are more active in the different subtypes of cluster headache. The difference between ECH and CCH is also suggested by the treatment response to a CGRP monoclonal antibody, galcanezumab (43,44), and non-invasive vagal nerve stimulator (45,46).

### Limitations

The main limitations to the study were the recruitment of eligible subjects and the dropout rate at each stage due to the nature of the condition. Therefore, a pragmatic approach was taken with the study design regarding randomisation. Although there was a randomisation sequence, this was modified by the investigator if deemed necessary. This would be in the case of episodic cluster headache patients that were near the end of their bout missing the NTG visit if they followed the randomised sequence. For this reason, it was designed to be a single-blind cross-over study and not a double-blind and randomised study. Expectation bias was reduced by maintaining blinding of the subjects until the end of the study and having both infusions identical in volume, appearance and duration of infusion. Furthermore, given the stark difference between placebo and NTG, there was no difference between attack onset in the open unblinded compared with the single-blinded NTG visits (Figure 4(b)), is an indication that randomisation was not a factor in this study.

## Conclusion

We present the results of the first placebo-controlled study using intravenous NTG administration to systematically study NTG-induced cluster headache attacks. We have shown that NTG can reliably bring on cluster headache attacks as well as the development of non-headache symptoms and CAS. This study highlights differences between NTG-induced attack onset times between the ECH and CCH subjects. Understanding the stages of cluster headache is essential; by recognising the non-headache symptoms in the lead up to the onset of pain and the underlying pathogenesis of this, we may be able to uncover new therapeutic targets to abort attacks before the onset of the severe and devastating pain experienced by our cluster headache patients.

## Article highlights

- Weight-calculated standardised intravenous nitroglycerin administration is a reliable method to induce cluster headache attacks, cranial autonomic symptoms and non-headache symptoms that accompany cluster headache attacks.
- Accompanying non-headache symptoms in cluster headache attacks are important to recognise for both clinical and research purposes.
- There may be inherent differences between episodic cluster headache and chronic cluster headache.
